# Detection of monoclonal protein by capillary zone electrophoresis can be challenged by iodinated contrast agent interference: a case report

**DOI:** 10.11613/BM.2021.021001

**Published:** 2021-04-15

**Authors:** Clément Capaldo, Mourad Cheddad El Aouni, Damien Laurelli, Cyril Leven, Jean-Luc Carré

**Affiliations:** 1 Department of Biochemistry and Pharmaco-Toxicology, University Hospital of Brest, Brest, France; 2 Neuroradiology Unit, Department of Radiology, University Hospital of Brest, Brest, France; 3 Univ Brest, EA 3878, GETBO, Brest, France; 4 Univ Brest, EA 4685, LIEN, Brest, France

**Keywords:** capillary electrophoresis, interference, iodinated contrast, gammopathy, case report

## Abstract

The detection of monoclonal immunoglobulins is a key element in the diagnosis of monoclonal gammopathy. In clinical practice, screening and measurement of monoclonal proteins are commonly performed using capillary zone electrophoresis (CZE). Some exogenous substances, such as iodinated contrast agents, absorb incident UV light at the same wavelengths as the peptide bonds and may therefore interfere with the detection of proteins in CZE. We herein use the description of a case to illustrate that iodinated contrast agents can mask the presence of monoclonal immunoglobulins in CZE and we discuss the strategy needed to confirm this interference. Performing immunofixation, immunosubtraction, or a second CZE at a distance from the first blood sample is not only necessary to confirm the presence of an iodinated contrast media interference but also to ensure the absence of monoclonal proteins.

## Introduction

The detection of monoclonal proteins remains essential in the management of monoclonal gammopathy for diagnosis, risk stratification, therapeutic assessment, and monitoring of disease progression (*1*). In clinical practice, screening and measurement of monoclonal proteins are commonly performed using capillary zone electrophoresis (CZE). Some exogenous substances, such as iodinated contrast agents, absorb incident UV light at the same wavelengths as the peptide bonds and may therefore interfere with the detection of proteins in CZE (*2*). Iodinated contrast agents show a supernumerary peak in β- and α-globulin fraction using CZE which may erroneously suggest the presence of monoclonal immunoglobulin (*3*). We herein use the description of a case to illustrate the need to explore interferences with iodinated contrast agents in order not to ignore the presence of monoclonal immunoglobulin.

## Case Report

**Figure 1:**
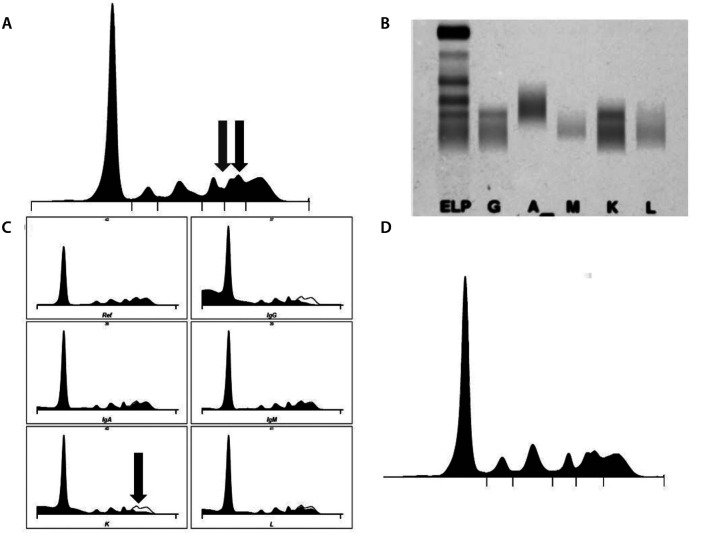
Results of capillary zone electrophoresis (A), immunofixation (B) and immunosubtraction (C) collected 8 hours after injection of iomeprol. Results of capillary zone electrophoresis collected 6 days after injection of iomeprol (D).

We report the case of a 90-year-old woman with no haematological history who was admitted to a neurovascular intensive care unit for suspected stroke. The patient had a left sensory-motor deficit and speech impairment. The blood sample was taken 8 hours after a computed tomography angiography and thrombectomy. Serum protein electrophoresis was performed on CAPILLARYS 2 (Sebia, Lisses, France). A duplication of the β-2 globulin fraction (black arrow) with an increase in concentration to 7.4 g/L (reference interval: 2.3-4.7 g/L) and a shoulder of β-1 globulin (grey arrow) were detected compared to normal serum (Figure 1A). Other laboratory results were unremarkable (Table 1). An informed consent form for the publication of a case report was signed by the patient during hospitalization.

**Table 1:** Laboratory findings at first admission

**Parameter (unit)**	**Result**	**Reference interval**
Creatinine (µmol/L)	33	40-66
Haemoglobin (g/L)	131	120-160
Mean cell volume (fL)	89	82-98
Calcium (mmol/L)	2.07	2.08-2.65
Albumin (g/L)	35	34-50
Total protein (g/L)	64	57-82
IgG (g/L)	13.0	6.1-13.0
IgA (g/L)	3.2	0.4-3.5
IgM (g/L)	1.2	0.5-3.0
IgG – immunoglobulin G. IgA – immunoglobulin A. IgM – immunoglobulin M.		

## Further investigations

**Figure 2:**
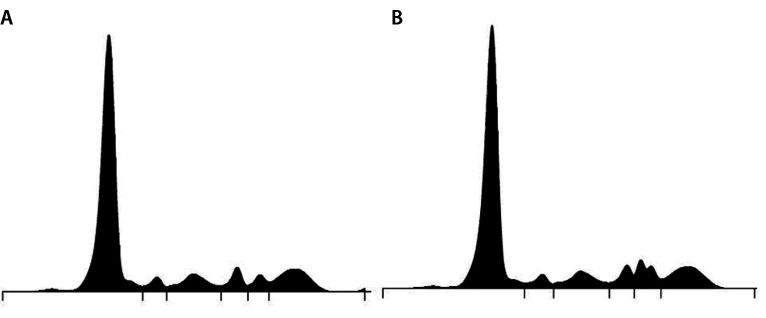
Results of capillary electrophoresis on normal serum concentrations (A) and normal serum spiked with iomeprol (B).

To explain the duplication of the β-2 globulin fraction, we hypothesized that we detect the radioopaque agent that was administered during the angiography (iomeprol) as previously reported (*4*). We performed immunofixation (HYDRAGEL, SEBIA, France) and immunosubtraction (CAPILLARYS 2, SEBIA, France) to test this hypothesis. IgG Kappa immunoglobulin was detected through immunofixation (Figure 1B) and immunosubtraction (Figure 1C) in the β-2 globulin fraction. Second protein electrophoresis (Figure 1D), performed on a sample taken 6 days after iomeprol injection, showed no β-1 globulin shoulder as opposed to the electrophoresis of the first sample, suggesting interference with the iodinated contrast agent. The absence of reaction with anti-IgG, -IgA, -IgM, -κ, -λ antibodies to the β-1 globulin fraction on immunosubtraction (Figure 1C) and immunofixation (Figure 1B) supported the presence of interference. To confirm the location of the iomeprol peak following electrophoresis on the CAPILLARYS 2 analyser, iomeprol was added to normal serum concentrations (Figure 2). The migration zone of iomeprol on the CAPILLARYS 2 analyser matched the location of the β1-globulin shoulder observed on the first electrophoresis (Figure 1A).

## What happened?

Finally, further analysis demonstrated that the duplication of the β-2 globulin fraction corresponded to monoclonal IgG Kappa and that te shoulder of the β-1 globulin fraction was due to interference by iomeprol.

## Discussion

Interference with iodinated contrast media is generally described as responsible for the appearance of false positive peaks in CZE (*5*). This observation shows that such interference can also mask the possible presence of monoclonal immunoglobulin. Indeed, monoclonal proteins and iodinated contrast agents can migrate to the same electropherogram fraction using capillary zone electrophoresis. On the one hand, in a cohort of 1027 myeloma multiple patients, the monoclonal protein migration was distributed in 12% in the β-fraction and 1% in the α-2 fraction (*6*). On the other hand, Arranz-Pena *et al.* described that a number of radio-opaque agents cause interference with the peak in the α2-globulin fraction, β-fraction, and pre-albumin (*3*). Further investigation is required to confirm the suspicion of interference so that the presence of monoclonal proteins at the CZE is not ignored. Interference with the radio-opaque product is easily confirmed by the absence of a monoclonal abnormality on immunofixation, immunosubtraction or by the disappearance of the supernumerary peak on a later sample for CZE analysis. Since the clearance of iodinated contrast agents is essentially renal, the size of the supernumerary peak depends on the patient’s renal function and the time between sampling and imaging (*7*). It is also possible to optimize the blood collection protocol to prevent interference and avoid further unnecessary studies by spacing intervals of 24 hours between blood sampling and the image examination, or 48 hours in case of renal failure (*8*).

In our hospital, the incidence of suspected interferences with iodinated contrast agents was low in 2019 (3.2‰ of the sample submitted for CZE) and was comparable to other centres (*8*, *9*). All suspicions of interference were tested or signalled to the clinicians associated with the recommendation to test the hypothesis by performing a second sampling.

In this case and according to the recommendations of the International Myeloma Working Group (IMWG), monoclonal gammopathy of undetermined significance (MGUS) was diagnosed in view of the monoclonal protein concentration < 30 g/L and the absence of organ damage due to plasma cell proliferation (hypercalcaemia, renal failure, anaemia, bone damage) (*1*). A myelogram was not proposed due to the advanced age of the patient and the poor expected benefits.

In conclusion, we would like to remind that iodinated contrast agents can mask the presence of monoclonal immunoglobulins migrating into the β- and α-globulin fraction using CZE. Performing immunofixation, immunosubtraction, or a second CZE at a distance from the first blood sample. Not only is necessary to confirm the presence of iodinated contrast media interference but also to ensure the absence of monoclonal proteins.

## What you should / can do in your laboratory to prevent such errors

Always perform further investigation in case of suspicion of interference with iodinated contrast agents.
Interferences can be highlighted by immunofixation, immunosubtraction or a second CZE at a distance from the first blood sample.
